# Supplementary material for: A Likelihood Approach for Real-Time Calibration of Stochastic Compartmental Epidemic Models
Source: PLoS Comput Biol. 2017 Jan 17;13(1):e1005257. doi: 10.1371/journal.pcbi.1005257 (PMC5240920; doi:10.1371/journal.pcbi.1005257)
Supplement: S1 File — (TAR.GZ) [file pcbi.1005257.s014.tar.gz › HSPH_Online-SI-Revision/Additions/PF09/n100-extreme_noisy/n100-extreme_noisy_PF_table0.pdf]

1	R0	{2.76052, 16.3913}	{2.08412, 17.995}	{{2.06644, 17.673}, {2.07961, 17.9841}}	{24.5026, 9.78393}	inf	26 580.	15 943.	{15 657.2, 15 972.7}	40.0187	0.222211	newly	10 934.9	6514.53	{6407., 6623.}	40.4243	0.224972	Reff	0.831027	1.30092	{1.29219, 1.30655}	56.5439	ar	92 090.	86 701.1	{86 021., 86 977.}	5.85176
2	R0	{2.18202, 15.1734}	{2.2005, 20.}	{{2.19209, 20.}, {2.20619, 20.}}	{0.846897, 31.8096}	inf	17 999.	20 825.9	{20 693.9, 20 954.9}	15.7058	0.0633489	newly	8027.	7247.06	{7154., 7348.}	9.71646	0.0444198	Reff	0.873157	1.01326	{1.00936, 1.01665}	16.0452	ar	84 264.	95 061.5	{94 442., 95 794.}	12.8139
3	R0	{2.57891, 12.5971}	{2.95839, 20.}	{{2.93379, 20.}, {2.97296, 20.}}	{14.7148, 58.7667}	inf	22 711.	30 709.	{30 540.4, 30 817.7}	35.2164	0.131027	newly	11 421.	10 418.7	{10 301., 10 546.}	8.7762	0.0399099	Reff	0.727149	0.91498	{0.912336, 0.917278}	25.8311	ar	90 302.	98 576.9	{98 353., 98 807.}	9.16358
4	R0	{2.5856, 9.28508}	{3., 20.}	{{3., 20.}, {3., 20.}}	{16.0272, 115.399}	inf	23 144.	31 572.3	{31 398., 31 726.2}	36.4166	0.134864	newly	15 345.	11 453.	{11 311., 11 585.}	25.3636	0.127068	Reff	0.760942	1.34832	{1.34448, 1.35193}	77.1911	ar	90 312.	103 504.	{103 097., 103 942.}	14.6068
5	R0	{2.4406, 12.1341}	{2.85502, 20.}	{{2.84858, 20.}, {2.85373, 20.}}	{16.9803, 64.8248}	inf	22 895.	28 466.6	{28 338.2, 28 525.5}	24.3353	0.0945875	newly	12 782.	10 216.8	{10 083., 10 347.}	20.0688	0.097307	Reff	0.972189	1.25811	{1.25476, 1.26542}	29.4103	ar	88 597.	97 669.4	{97 458., 97 932.}	10.2401
6	R0	{2.72155, 15.4094}	{2.17909, 16.7429}	{{2.17644, 16.7099}, {2.18147, 16.7916}}	{19.932, 8.65404}	inf	26 147.	17 444.9	{17 395.9, 17 481.7}	33.2816	0.175756	newly	11 271.	7651.84	{7546., 7736.}	32.1104	0.168214	Reff	0.880857	1.2981	{1.2959, 1.3003}	47.3678	ar	91 682.	88 811.1	{88 574., 89 019.}	3.1314
7	R0	{2.63781, 14.3935}	{2.12977, 20.}	{{2.12163, 20.}, {2.13388, 20.}}	{19.2598, 38.9516}	inf	24 939.	15 658.5	{15 526.3, 15 749.1}	37.2127	0.202136	newly	11 237.	5847.36	{5747., 5933.}	47.9633	0.283722	Reff	0.790499	1.41601	{1.41495, 1.41703}	79.1289	ar	91 192.	89 513.3	{89 079., 89 834.}	1.84085
8	R0	{2.82391, 16.104}	{2.14445, 16.6776}	{{2.14221, 16.6479}, {2.14658, 16.7228}}	{24.0608, 3.56195}	inf	27 368.	16 991.8	{16 943.9, 17 048.6}	37.9135	0.207006	newly	11 207.	7457.07	{7352., 7578.}	33.4606	0.176948	Reff	0.861801	1.2793	{1.27717, 1.28131}	48.445	ar	92 664.	87 297.9	{86 959., 87 615.}	5.79093
9	R0	{2.80645, 14.6457}	{2.12844, 17.3042}	{{2.12732, 17.2772}, {2.12923, 17.3291}}	{24.1588, 18.152}	inf	27 606.	15 465.2	{15 441.7, 15 491.9}	43.9789	0.251648	newly	12 694.	6702.22	{6609., 6804.}	47.2017	0.277406	Reff	0.94945	1.40378	{1.40246, 1.40488}	47.8514	ar	92 567.	88 487.8	{88 218., 88 755.}	4.40672
10	R0	{1.86213, 5.4271}	{3., 20.}	{{3., 20.}, {3., 20.}}	{61.1058, 268.521}	inf	10 896.	32 695.4	{32 523.4, 32 827.4}	200.068	0.477217	newly	11 765.	11 564.8	{11 430., 11 700.}	1.7054	0.00748569	Reff	0.763157	1.18956	{1.18639, 1.19207}	55.8732	ar	75 362.	103 036.	{102 704., 103 370.}	36.722
11	R0	{2.27349, 14.6338}	{2.22456, 17.7443}	{{2.21942, 17.6447}, {2.22994, 17.8356}}	{2.15227, 21.2553}	inf	19 721.	20 574.3	{20 449.4, 20 706.9}	4.3268	0.0183921	newly	8893.	8043.31	{7938., 8145.}	9.55459	0.0436343	Reff	0.854264	1.01542	{1.00971, 1.0209}	18.8651	ar	85 805.	92 643.7	{92 309., 92 907.}	7.97007
12	R0	{2.77261, 14.1894}	{2.2213, 20.}	{{2.21478, 20.}, {2.22771, 20.}}	{19.8843, 40.9503}	inf	27 138.	16 253.8	{16 169.6, 16 311.3}	40.1069	0.222626	newly	12 745.	6133.04	{6043., 6221.}	51.8789	0.317693	Reff	0.865526	1.48805	{1.48655, 1.48955}	71.9249	ar	92 508.	91 386.7	{91 029., 91 708.}	1.21206
13	R0	{2.65402, 19.3935}	{2.1963, 20.}	{{2.19082, 20.}, {2.19934, 20.}}	{17.2464, 3.12734}	inf	25 765.	15 652.5	{15 515.6, 15 740.3}	39.2491	0.216454	newly	8948.	5702.2	{5598., 5807.}	36.274	0.195728	Reff	0.987375	1.28277	{1.28146, 1.28523}	29.9169	ar	91 184.	91 623.5	{91 282., 91 978.}	0.493815
14	R0	{1.75297, 4.45354}	{2.18388, 17.8894}	{{2.17925, 17.8148}, {2.18746, 17.9733}}	{24.5816, 301.689}	inf	10 727.	10 377.5	{10 309.7, 10 434.8}	3.25779	0.0143884	newly	15 310.	4697.57	{4609., 4784.}	69.317	0.513149	Reff	0.926059	1.78174	{1.77922, 1.78481}	92.4	ar	71 263.	90 528.	{90 262., 90 803.}	27.0337
15	R0	{1.99928, 7.1501}	{2.90813, 20.}	{{2.90142, 20.}, {2.91616, 20.}}	{45.4587, 179.716}	inf	13 996.	29 230.4	{28 965.9, 29 598.1}	108.848	0.319811	newly	12 056.	10 381.7	{10 213., 10 576.}	13.8875	0.0649713	Reff	0.80563	1.19848	{1.1943, 1.20433}	48.7628	ar	79 602.	99 084.3	{98 383., 99 626.}	24.4746
16	R0	{2.67398, 10.3134}	{2.94282, 20.}	{{2.94066, 20.}, {2.94504, 20.}}	{10.0539, 93.9225}	inf	23 578.	29 134.2	{28 954.4, 29 206.1}	23.5652	0.0918943	newly	14 011.	10 630.4	{10 500., 10 742.}	24.128	0.119935	Reff	0.705449	1.37106	{1.36983, 1.3726}	94.3521	ar	91 289.	100 139.	{99 889., 100 374.}	9.69476
17	R0	{2.44634, 16.3226}	{2.21392, 20.}	{{2.21096, 20.}, {2.21648, 20.}}	{9.50079, 22.5295}	inf	22 666.	20 745.8	{20 654.3, 20 841.2}	8.47156	0.0384481	newly	9419.	7336.01	{7231., 7432.}	22.1148	0.108567	Reff	0.907103	1.11615	{1.11411, 1.11929}	23.0457	ar	88 710.	94 549.4	{94 172., 94 849.}	6.58254
18	R0	{2.02168, 10.1676}	{2.11248, 20.}	{{2.10994, 20.}, {2.11454, 20.}}	{4.4912, 96.7033}	inf	15 535.	11 684.9	{11 646., 11 720.1}	24.7832	0.123687	newly	10 392.	4387.88	{4313., 4456.}	57.7764	0.374482	Reff	0.996001	1.44345	{1.44248, 1.44456}	44.9246	ar	80 299.	87 372.7	{87 034., 87 687.}	8.80919
19	R0	{2.89969, 15.009}	{2.22938, 20.}	{{2.2179, 20.}, {2.23372, 20.}}	{23.1166, 33.2534}	inf	28 996.	16 252.7	{16 100.1, 16 345.4}	43.9485	0.251421	newly	12 862.	6145.72	{6048., 6226.}	52.218	0.320762	Reff	0.881738	1.50527	{1.50328, 1.50725}	70.7164	ar	93 349.	91 773.	{91 434., 92 069.}	1.68827
20	R0	{2.68933, 7.34928}	{3., 20.}	{{3., 20.}, {3., 20.}}	{11.5519, 172.136}	inf	24 920.	22 767.	{22 682.2, 22 838.6}	8.63965	0.039244	newly	20 039.	9172.63	{9065., 9291.}	54.2261	0.339403	Reff	0.765733	1.94732	{1.945, 1.94969}	154.308	ar	91 574.	100 493.	{100 214., 100 801.}	9.74002
21	R0	{2.15352, 9.28074}	{2.94488, 20.}	{{2.94241, 20.}, {2.94712, 20.}}	{36.7474, 115.5}	inf	17 876.	29 575.3	{29 524.4, 29 613.9}	65.447	0.218658	newly	12 361.	10 486.2	{10 394., 10 579.}	15.1672	0.0714472	Reff	0.8829	1.19016	{1.18845, 1.19126}	34.8015	ar	83 845.	99 509.1	{99 384., 99 686.}	18.6822
22	R0	{2.21953, 9.04387}	{3., 20.}	{{3., 20.}, {3., 20.}}	{35.1638, 121.144}	inf	18 399.	33 391.7	{33 262.3, 33 522.}	81.4864	0.258842	newly	13 126.	11 609.6	{11 476., 11 718.}	11.5523	0.0533297	Reff	0.851523	1.07833	{1.07219, 1.08924}	26.635	ar	84 856.	102 339.	{101 967., 102 640.}	20.6037
23	R0	{2.21987, 14.5347}	{1.95177, 20.}	{{1.95049, 20.}, {1.9529, 20.}}	{12.0772, 37.6017}	inf	18 816.	13 485.4	{13 444.4, 13 519.2}	28.3299	0.144663	newly	8717.	4939.47	{4859., 5008.}	43.3352	0.246718	Reff	0.868968	1.29075	{1.28998, 1.29197}	48.5379	ar	85 067.	83 907.5	{83 595., 84 210.}	1.36303
24	R0	{2.84268, 17.9659}	{1.95391, 20.}	{{1.95223, 20.}, {1.95562, 20.}}	{31.2654, 11.322}	inf	27 580.	12 907.6	{12 865.7, 12 963.6}	53.1994	0.329752	newly	10 377.	4756.99	{4669., 4843.}	54.1583	0.338779	Reff	0.920602	1.32672	{1.32564, 1.32791}	44.1139	ar	92 836.	83 606.7	{83 282., 83 973.}	9.94149
25	R0	{2.52706, 10.3679}	{2.10661, 20.}	{{2.09841, 20.}, {2.11013, 20.}}	{16.638, 92.9031}	inf	22 248.	12 601.7	{12 504.5, 12 659.5}	43.3581	0.246867	newly	13 481.	4830.83	{4741., 4913.}	64.1656	0.445738	Reff	0.761075	1.56808	{1.56513, 1.57082}	106.035	ar	89 623.	87 904.5	{87 514., 88 332.}	1.91744
26	R0	{2.62835, 19.1088}	{2.27861, 20.}	{{2.27332, 20.}, {2.28306, 20.}}	{13.3066, 4.66382}	inf	24 689.	22 128.6	{21 964.4, 22 232.4}	10.3705	0.047553	newly	8698.	7745.93	{7620., 7863.}	10.9458	0.0503728	Reff	0.928833	1.05798	{1.05453, 1.06096}	13.904	ar	90 908.	96 354.2	{95 989., 96 663.}	5.99089
27	R0	{2.76831, 19.4155}	{2.15561, 16.9833}	{{2.14611, 16.8782}, {2.15855, 17.0354}}	{22.1328, 12.5272}	inf	25 708.	18 964.5	{18 838.2, 18 985.}	26.2311	0.132153	newly	8776.	7709.22	{7618., 7810.}	12.1557	0.0563085	Reff	0.75287	0.983099	{0.978127, 0.989794}	30.5802	ar	92 175.	89 380.2	{89 110., 89 684.}	3.03209
28	R0	{2.96609, 16.8903}	{3., 20.}	{{3., 20.}, {3., 20.}}	{1.14326, 18.4112}	inf	28 896.	31 739.6	{31 558.7, 31 897.2}	9.84064	0.0407599	newly	11 093.	10 663.3	{10 537., 10 786.}	3.87406	0.0171785	Reff	0.743925	0.849208	{0.838207, 0.862219}	14.1524	ar	94 024.	100 476.	{100 059., 100 859.}	6.8626
29	R0	{2.17046, 14.39}	{1.98132, 20.}	{{1.97655, 20.}, {1.98446, 20.}}	{8.71426, 38.9854}	inf	18 683.	13 795.6	{13 708.6, 13 856.6}	26.1594	0.131708	newly	8778.	5067.22	{4976., 5142.}	42.2736	0.238664	Reff	0.946993	1.30864	{1.30708, 1.31044}	38.1884	ar	84 001.	85 462.4	{85 117., 85 840.}	1.73978
30	R0	{2.88772, 12.4647}	{3., 20.}	{{3., 20.}, {3., 20.}}	{3.88819, 60.4531}	inf	28 167.	30 770.1	{30 644.4, 30 855.4}	9.24178	0.0383874	newly	14 527.	11 188.5	{11 062., 11 293.}	22.9816	0.113421	Reff	0.836804	1.35889	{1.35711, 1.36125}	62.3901	ar	93 273.	101 521.	{101 263., 101 768.}	8.84284
31	R0	{2.46831, 8.14308}	{3., 20.}	{{3., 20.}, {3., 20.}}	{21.5406, 145.607}	inf	22 336.	28 261.6	{28 126.5, 28 371.}	26.5295	0.102189	newly	17 282.	10 778.	{10 621., 10 903.}	37.6345	0.205077	Reff	0.883087	1.64452	{1.64044, 1.6478}	86.2235	ar	88 762.	103 362.	{102 892., 103 739.}	16.4485
32	R0	{1.93236, 7.38987}	{3., 20.}	{{3., 20.}, {3., 20.}}	{55.2506, 170.641}	inf	13 853.	32 224.1	{32 108.8, 32 294.7}	132.615	0.366636	newly	12 032.	11 151.9	{11 041., 11 260.}	7.31458	0.0330036	Reff	0.884615	1.04684	{1.04147, 1.05116}	18.3389	ar	77 654.	100 876.	{100 590., 101 100.}	29.9051
33	R0	{2.82251, 17.6954}	{2.89003, 20.}	{{2.85638, 20.}, {2.9256, 20.}}	{2.39202, 13.0237}	inf	27 972.	29 928.2	{29 440.6, 30 474.9}	6.99352	0.0293251	newly	10 722.	10 393.5	{10 206., 10 570.}	3.0693	0.0135765	Reff	1.00304	1.06025	{1.03673, 1.09082}	5.70453	ar	92 745.	98 120.9	{97 702., 98 453.}	5.79641
34	R0	{1.87083, 12.3994}	{2.25752, 20.}	{{2.24212, 20.}, {2.27061, 20.}}	{20.6696, 61.2981}	inf	12 842.	18 034.6	{17 704.9, 18 273.1}	40.4346	0.147443	newly	7135.	6403.28	{6271., 6521.}	10.2554	0.0470406	Reff	1.01449	1.13815	{1.12987, 1.14573}	12.1885	ar	76 282.	94 453.4	{93 860., 94 985.}	23.8213
35	R0	{2.95779, 16.3003}	{2.17276, 16.2354}	{{2.16948, 16.216}, {2.17449, 16.2606}}	{26.5411, 0.397893}	inf	29 124.	15 918.6	{15 864.2, 15 962.3}	45.3421	0.262348	newly	12 158.	7375.8	{7285., 7463.}	39.3338	0.217071	Reff	1.01659	1.40318	{1.40193, 1.40464}	38.0282	ar	93 873.	87 960.	{87 676., 88 215.}	6.29897
36	R0	{2.18718, 14.261}	{2.17752, 16.1898}	{{2.16662, 16.089}, {2.18463, 16.2753}}	{0.442977, 13.5248}	inf	18 672.	19 263.3	{19 093.9, 19 430.5}	3.16671	0.0135291	newly	8858.	8242.97	{8116., 8387.}	6.94322	0.0312888	Reff	0.915641	1.01194	{1.00144, 1.02226}	10.517	ar	84 379.	88 511.7	{88 113., 88 874.}	4.89783
37	R0	{2.44912, 9.31118}	{3., 20.}	{{3., 20.}, {3., 20.}}	{22.493, 114.796}	inf	21 427.	31 597.4	{31 473.8, 31 726.3}	47.4652	0.168688	newly	14 344.	11 239.4	{11 117., 11 366.}	21.6438	0.105944	Reff	0.796601	1.22324	{1.22081, 1.22621}	53.5577	ar	88 442.	102 152.	{101 831., 102 453.}	15.5018
38	R0	{2.05949, 5.72514}	{3., 20.}	{{3., 20.}, {3., 20.}}	{45.6671, 249.336}	inf	16 011.	25 621.4	{25 492., 25 717.8}	60.0235	0.204181	newly	16 782.	9971.24	{9853., 10 088.}	40.5837	0.226112	Reff	0.827297	1.7538	{1.7509, 1.75735}	111.991	ar	81 587.	100 752.	{100 450., 101 014.}	23.4908
39	R0	{1.75505, 8.52404}	{2.34141, 20.}	{{2.32129, 20.}, {2.35024, 20.}}	{33.4096, 134.631}	inf	10 952.	22 616.5	{22 331.3, 22 843.4}	106.506	0.314914	newly	8590.	8082.88	{7915., 8217.}	5.90361	0.026464	Reff	0.957748	1.18879	{1.18556, 1.19322}	24.1234	ar	70 925.	97 417.9	{96 982., 97 893.}	37.3533
40	R0	{2.24724, 13.7861}	{2.30672, 17.2846}	{{2.2814, 17.0688}, {2.33216, 17.4769}}	{2.64838, 25.3771}	inf	18 516.	23 844.8	{23 334.2, 24 320.2}	28.7793	0.109799	newly	8935.	9434.99	{9231., 9635.}	5.59586	0.0235846	Reff	0.839861	0.952161	{0.939225, 0.965347}	13.3713	ar	85 257.	96 827.6	{95 974., 97 537.}	13.5714
41	R0	{1.77635, 8.62406}	{2.35568, 16.6578}	{{2.07829, 13.8037}, {2.7207, 20.}}	{32.6137, 93.1546}	inf	11 281.	23 750.6	{19 408.8, 29 529.2}	110.537	0.316035	newly	8585.	9736.22	{9447., 10 118.}	13.4097	0.0544899	Reff	0.87725	0.964819	{0.930101, 0.993719}	10.0039	ar	72 941.	96 401.6	{92 253., 101 479.}	32.1638
42	R0	{1.80397, 5.4932}	{3., 20.}	{{3., 20.}, {3., 20.}}	{66.2999, 264.087}	inf	10 692.	32 134.1	{31 971.7, 32 259.9}	200.543	0.477905	newly	11 817.	11 403.4	{11 272., 11 527.}	3.50021	0.0154905	Reff	0.8332	1.20696	{1.2041, 1.21107}	44.8586	ar	73 232.	101 442.	{101 154., 101 689.}	38.5212
43	R0	{2.83096, 16.7077}	{1.90926, 20.}	{{1.90789, 20.}, {1.90957, 20.}}	{32.5578, 19.7053}	inf	27 789.	9913.61	{9893.42, 9927.89}	64.3254	0.447642	newly	11 209.	3750.63	{3684., 3819.}	66.5391	0.475512	Reff	0.932207	1.46856	{1.4674, 1.46945}	57.5362	ar	92 740.	82 213.5	{81 772., 82 595.}	11.3505
44	R0	{2.73678, 11.6021}	{3., 20.}	{{3., 20.}, {3., 20.}}	{9.61787, 72.3826}	inf	23 763.	32 110.7	{31 984.7, 32 219.3}	35.1288	0.130746	newly	12 451.	11 083.8	{10 949., 11 196.}	10.981	0.0505336	Reff	0.638655	1.03143	{1.02894, 1.03444}	61.5002	ar	92 003.	100 283.	{100 035., 100 494.}	8.99951
45	R0	{2.14235, 6.98601}	{2.94262, 20.}	{{2.93604, 20.}, {2.94584, 20.}}	{37.3547, 186.286}	inf	15 339.	29 132.3	{28 977.5, 29 212.7}	89.9228	0.278574	newly	13 010.	10 594.7	{10 467., 10 701.}	18.565	0.0892051	Reff	0.718266	1.35537	{1.35353, 1.35833}	88.7006	ar	82 974.	99 536.1	{99 275., 99 728.}	19.9606
46	R0	{2.23409, 9.21289}	{2.84725, 20.}	{{2.84011, 20.}, {2.85122, 20.}}	{27.4455, 117.087}	inf	19 256.	27 173.9	{27 014.1, 27 275.7}	41.119	0.14958	newly	14 108.	10 025.3	{9896., 10 140.}	28.9389	0.148388	Reff	0.983357	1.4256	{1.42371, 1.42776}	44.9723	ar	85 163.	99 299.1	{99 023., 99 538.}	16.5988
47	R0	{2.12258, 8.15265}	{3., 20.}	{{3., 20.}, {3., 20.}}	{41.3374, 145.319}	inf	17 054.	32 151.5	{31 976.3, 32 383.6}	88.5279	0.27537	newly	13 477.	11 378.8	{11 241., 11 515.}	15.569	0.0735173	Reff	0.871829	1.19477	{1.18779, 1.20113}	37.0421	ar	82 690.	104 578.	{104 042., 105 076.}	26.4701
48	R0	{2.71771, 10.4636}	{3., 20.}	{{3., 20.}, {3., 20.}}	{10.3871, 91.1388}	inf	26 340.	30 105.4	{29 954.3, 30 226.8}	14.2955	0.0580257	newly	16 548.	11 209.4	{11 082., 11 340.}	32.2612	0.169183	Reff	0.927228	1.50389	{1.50139, 1.50703}	62.192	ar	91 716.	101 920.	{101 531., 102 250.}	11.1258
49	R0	{2.44369, 6.66477}	{3., 20.}	{{3., 20.}, {3., 20.}}	{22.7652, 200.085}	inf	18 124.	27 607.5	{27 446.6, 27 731.6}	52.3255	0.18277	newly	14 957.	10 597.1	{10 463., 10 736.}	29.1497	0.149678	Reff	0.592717	1.68463	{1.67986, 1.68826}	184.222	ar	88 827.	103 366.	{102 888., 103 758.}	16.3674
50	R0	{2.77383, 17.9775}	{2.16321, 20.}	{{2.16037, 20.}, {2.16485, 20.}}	{22.0136, 11.2502}	inf	25 584.	19 169.5	{19 064.9, 19 235.8}	25.0723	0.12536	newly	9271.	6846.77	{6770., 6932.}	26.1485	0.131665	Reff	0.747852	1.16776	{1.16648, 1.16969}	56.1479	ar	92 334.	91 643.5	{91 192., 92 038.}	0.747807
